# Novel and simple transformation algorithm for combining microarray data sets

**DOI:** 10.1186/1471-2105-8-218

**Published:** 2007-06-25

**Authors:** Ki-Yeol Kim, Dong Hyuk Ki, Ha Jin Jeong, Hei-Cheul Jeung, Hyun Cheol Chung, Sun Young Rha

**Affiliations:** 1 Oral Cancer Research Institute, Yonsei University College of Dentistry, Seoul, 120-752, Korea; 2 Cancer Metastasis Research Center, Yonsei University College of Medicine, Seoul, 120-752, Korea; 3 National Biochip Research Center, Yonsei University College of Medicine, Seoul, 120-752, Korea; 4 Brain Korea 21 Project for Medical Science, Yonsei University College of Medicine, Seoul, 120-752, Korea; 5 Yonsei Cancer Center, Yonsei University College of Medicine, Seoul, 120-752, Korea; 6 Department of Internal Medicine, Yonsei University College of Medicine, Seoul, 120-752, Korea

## Abstract

**Background:**

With microarray technology, variability in experimental environments such as RNA sources, microarray production, or the use of different platforms, can cause bias. Such systematic differences present a substantial obstacle to the analysis of microarray data, resulting in inconsistent and unreliable information. Therefore, one of the most pressing challenges in the field of microarray technology is how to integrate results from different microarray experiments or combine data sets prior to the specific analysis.

**Results:**

Two microarray data sets based on a 17k cDNA microarray system were used, consisting of 82 normal colon mucosa and 72 colorectal cancer tissues. Each data set was prepared from either total RNA or amplified mRNA, and the difference of RNA source between these two data sets was detected by ANOVA (Analysis of variance) model. A simple integration method was introduced which was based on the distributions of gene expression ratios among different microarray data sets. The method transformed gene expression ratios into the form of a reference data set on a gene by gene basis. Hierarchical clustering analysis, density and box plots, and mixture scores with correlation coefficients revealed that the two data sets were well intermingled, indicating that the proposed method minimized the experimental bias. In addition, any RNA source effect was not detected by the proposed transformation method. In the mixed data set, two previously identified subgroups of normal and tumor were well separated, and the efficiency of integration was more prominent in tumor groups than normal groups. The transformation method was slightly more effective when a data set with strong homogeneity in the same experimental group was used as a reference data set.

**Conclusion:**

Proposed method is simple but useful to combine several data sets from different experimental conditions. With this method, biologically useful information can be detectable by applying various analytic methods to the combined data set with increased sample size.

## Background

DNA microarrays are a useful tool for the study of complex systems and have applications in a wide variety of biological sciences. Despite their usefulness, however, systematic biases caused by different handling procedures present a challenge. Microarray experiments are often performed over many months, and samples are often collected and processed at different institutions. Further, the samples may be assayed using different microarray print batches or platforms, or using different array hybridization protocols. When two microarray data sets are directly compared, systematic biases arising from variability in experimental conditions can be erroneously detected as differences in gene expression patterns. Such systemic biases present a substantial obstacle in the analysis of microarray data. However, due to the limited numbers of available microarray experiments, the motivation to use an entire data set regardless of platforms or experimental procedure is increasing. Therefore, it is necessary to investigate new methods that can effectively combine microarray data sets which were derived from different experimental environments, while simultaneously minimizing systematic bias.

A commonly utilized method to integrate microarray data sets is to focus on the differential expression, i.e. comparing significantly expressed genes selected separately from each data set [1-7]. Another type of comparison examines the variability in gene expressions between human and mouse data sets combining the different microarray platforms [4]. These studies exploit multiple data sets, rather than a single data set, in order to obtain more robust result. Some studies overcome the limitations of a single microarray data set using integration technique, since integration of separate data sets has the similar effect as increasing sample size [8]. However, a suitable integration method has not yet been established. Indeed, some studies suggest that microarray data sets derived from different experimental processes cannot be combined directly, as they are poorly correlated with each other [9].

Recently, the practice of integrating data sets prior to selecting significant genes was introduced and standardization has been used for this as the simplest method [10]. Singular Value Decomposition (SVD) corrects systematic bias of data sets and has been used in yeast cell cycle experiments [11] and in data sets containing samples from many soft tissue tumors [12]. Although SVD is a useful method for determining the direction of large variations so that systematic effects can be removed, it has been suggested that SVD is inappropriate for cases where the magnitude of the systematic variation is similar to the components of other variations [13]. Alternatively, Distance Weighted Discrimination (DWD), which is a modified form of SVM that adjusts for systematic effects, performed well and could eliminate source effects [13]. However, DWD could not regulate the dispersion of different data sets.

A method that transforms the distributions of gene expressions of two data sets similarly was proposed [14]. However, this method did not consider biological differences between the two different experimental groups, such as normal and tumor, because they used the average expression value of these two groups to define a reference sample. A recent study introduced an ANOVA, Analysis of Variance, model to select discriminative genes from several datasets derived from different experimental environments [15]. This method can be flexible to consider any clinical variables as well as genetic information including several effect factors, which represent experimental conditions. But, with this method, we can not evaluate how well datasets are intermixed, and explore expression patterns of any interesting genes in combined data set. Therefore, we suggested a method to effectively integrate different experimental environments and evaluated its efficiency using *mixture score*.

## Results

Seventy-eight experiments (43 tumor and 35 normal) from data set A and 76 experiments (39 tumor and 37 normal) from data set B were used in this study. The whole data set included missing entries in the range of 448 to 1298 genes for each experiment. A total of 12293 genes without missing entries were used for further analysis.

### Exploration of expression patterns of two data sets before transformation

**Figure 1:**
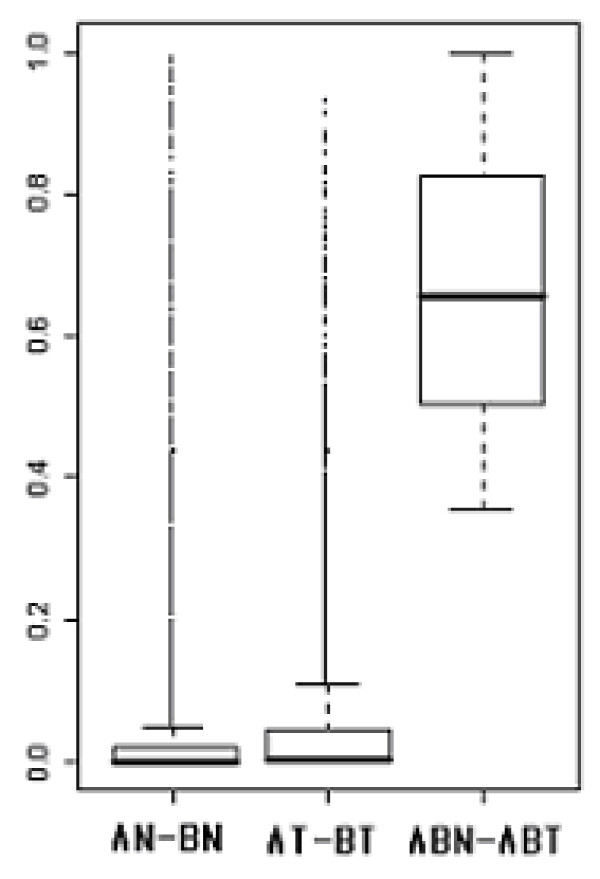
Comparison of the distributions of p-values for differently expressed genes based on two data sets. Two sample t-test was executed for selection of significantly differently expressed genes between two data sets. (AN-BN: normal groups of data set A and data set B, AT-BT: tumor groups of data set A and data set B, ABN-ABT: normal group and tumor group of combined data set without transformation)

We confirmed RNA source effect in more than 5000 genes by ANOVA using adjusted p-value of 0.05 by Bonferroni correction (data not shown). The numbers of genes which were significantly differentially expressed between two experimental groups were 2325 and 1654 in data set A and data set B, respectively. This showed that there was some difference in sensitivity between two data sets. Even in same experimental group, there were many genes that expressed differently between two data sets, 4668 genes in normal group and 3364 in tumor group. The significant difference between different experimental groups, normal and tumor groups, was not detected by RNA source effect in these genes. Figure 1 shows the distribution of p-values for differently expressed genes based on two data sets.

**Figure 2:**
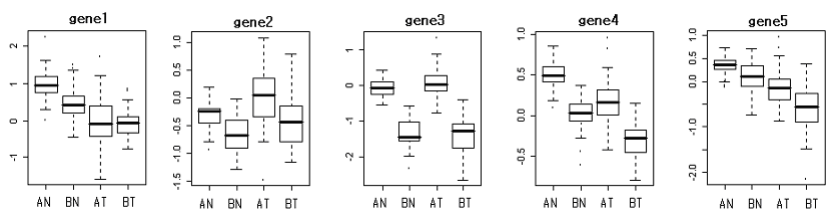
Boxplots for expression ratios of five randomly selected genes in normal and tumor groups. AN, AT, BN and BT are normal and tumor groups in data set A and data set B, respectively.

As shown in Figure 2, there were considerable differences in the scales and locations of expression ratios of five randomly selected genes from two data sets, even in the same group, prior to transformation of gene expression. Data set B seemed to have larger variation than data set A in expression ratios in both tumor and normal groups, and tumor groups had large dispersions in two data sets.

**Figure 3:**
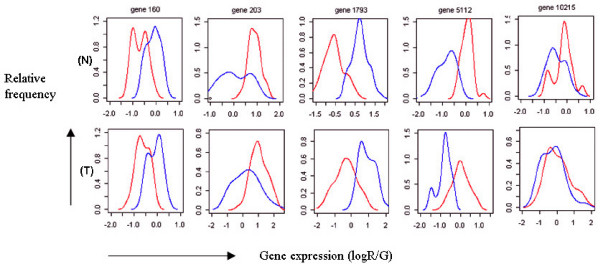
Density plots of five randomly selected genes from the data sets of (N) normal and (T) tumor groups (red: data set A, blue: data set B). Horizontal and vertical axes represent gene expression and relative frequency, respectively.

In analyzing the five randomly selected genes with density plots, we found significant differences in locations of the first four genes, and in dispersions of gene203 and gene5112 between the two data sets (Figure 3). If the data sets are analyzed without transformation, gene203 and gene5112 in data set B, whose variations of expression ratios were larger in data set B compared with data set A, is relatively more influential than the same genes of data set A in further analysis. Likewise, because of the large differences in locations of gene160 and gene1793 between the two data sets, these genes might not be selected as discriminative genes classifying the two different experimental groups.

**Figure 4:**
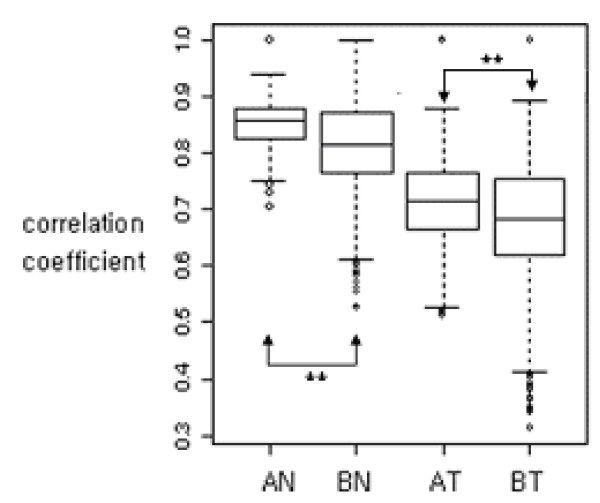
Comparison of within-group correlations (AN, BN, AT, BT: normal and tumor groups in data set A and data set B, respectively). ** P-value < 0.01.

Figure 4 shows that data set A had a stronger relationship within experimental groups compared to data set B, indicating that data set A is more homogeneous than data set B. However, the within-group correlations in tumor groups were lower than those within the normal groups in both data sets. Actually, average correlation coefficients within normal groups were 0.85 and 0.81, and 0.72 and 0.68 within tumor groups in data set A and data set B, respectively. This can be explained by the fact that the tumor groups showed larger variations than the normal groups, due to variability in biological conditions including different disease stages.

**Figure 5:**
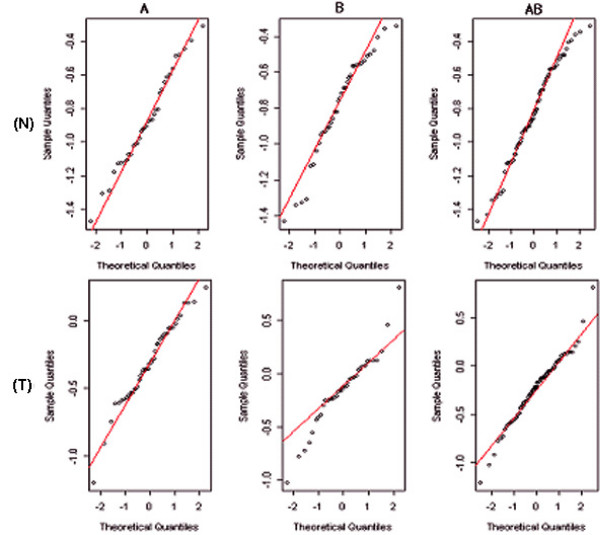
Q-Q plots of a randomly selected genes in (N) normal and (T) tumor groups. (A: data set A; B: data set B; AB: combined data sets)

As the normality assumption is necessary for applying t-test, Q-Q plot was evaluated. The two separate data sets did not satisfy the normality assumption in normal and tumor groups before combining two data sets as shown in Figure 5. This figure also indicates that data set B had larger variation than data set A for both tumor and normal groups. However, the normality in the combined data set was improved even though no transformation was applied to two data sets. It can be interpreted that the combined data set slightly follows a normal distribution due to the increased sample size resulting from the combining of the two data sets. It indicates that the integration of two data sets is essential for reliable result.

### Exploration of expression patterns of combined data sets after transformation

**Figure 6:**
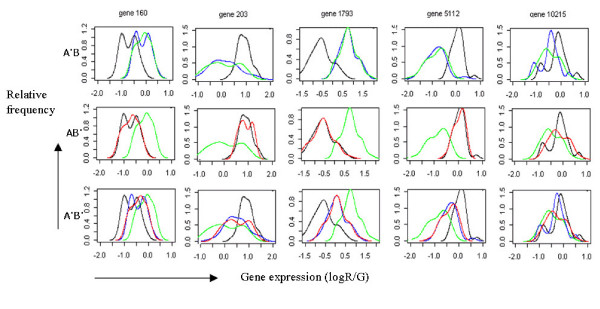
Comparison of integration methods which are A'B, AB' and A'B', using density plots of five randomly selected genes from the normal group (black: data set A, green: data set B, blue: transformed data set A in A'B and A'B', red: transformed data set B in AB' and A'B'). Horizontal and vertical axes represent gene expression values and relative frequency, respectively.

As explained in the "Methods", A'B transforms data set A referring to data set B and AB' transforms data set B referring to data set A. A'B' transforms data set A and data set B using pooled standard deviation and combines them. The variations observed in data sets transformed by the A'B and AB' methods changed depending on the variations of the reference data set (Figure 6). The A'B' method transformed expression patterns of the two data sets using a pooled standard deviation and corrected the difference in variations of them. We observed that the differences in location and variation between the two data sets were adjusted by the proposed methods preserving the respective expression patterns.

**Figure 7:**
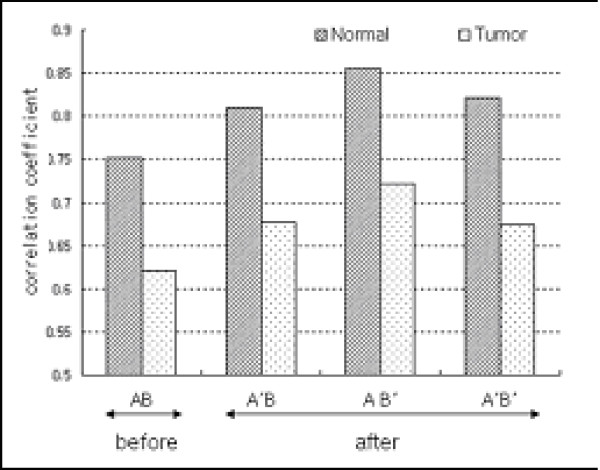
Comparison of within-group correlations. (AB: combined data set before transformation; A'B, AB', A'B': combined data sets after transformation)

Data set A had higher within-group correlation than data set B prior to integration, indicating that experiments in data set A had more homogeneous expression pattern than data set B (Figure 4). The tumor groups had lower correlations than the normal groups in both data sets, indicating that there were larger variations within the tumor groups than the normal groups. Within-group correlations of the combined data sets after transformation were increased comparing to the result shown in Figure 4 (Figure 7). The homogeneity of the combined data set A'B was similar to that of data set B, because data set A'B was resulted from the transformation of data set A into the expression pattern of data set B with low homogeneity within group. Similarly, data set AB' preserved relatively strong homogeneity, by transforming into expression patterns of data set A with higher correlation than data set B. Data set A'B', which was transformed by the weighted average of the dispersions of the two data sets, had an average degree of homogeneity of A'B and AB', but still had a low correlation in the tumor group. The data sets integrated by the proposed transformation methods had higher correlations within groups than the integrated data set without transformation, indicating that the proposed transformation methods effectively preserved homogeneity within groups of separate data sets.

**Figure 8:**
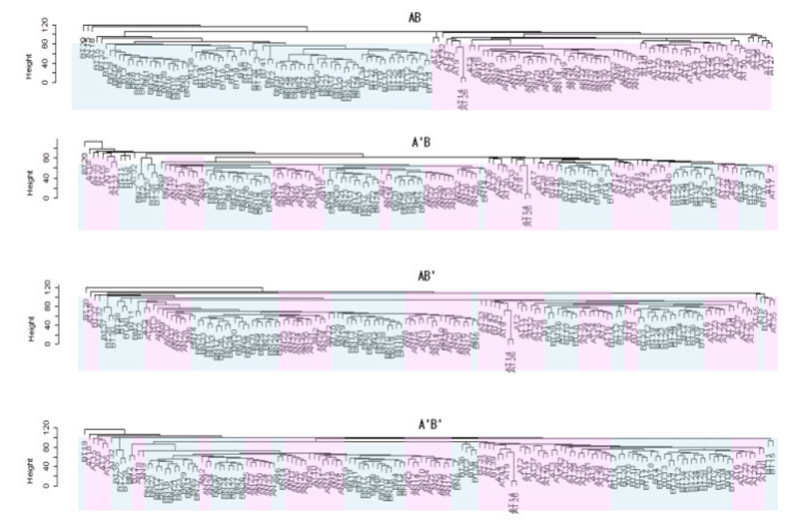
Comparison of integration methods which are AB, A'B, AB' and A'B', using unsupervised hierarchical cluster analysis. All of data sets include two experimental groups, normal and tumor. Euclidean distance and average linkage method were used as similarity measure and linkage method for hierarchical cluster analysis (pink: data set A; blue: data set B).

Hierarchical cluster analysis (HCA) clustered the samples into two distinct groups according to the data sources rather than different experimental groups (Figure 8AB). However, Figure 8A'B, AB' and 8A'B' showed that the experimental groups (tumor or normal) of the two data sets were more distinctly separated regardless of the transformation methods. HCA also showed that the two data sets were in fact well intermingled, indicating that the experimental bias had been minimized. Intermingling of different data sets indicates that data sets derived from different experimental conditions are combined well for further analysis.

**Figure 9:**
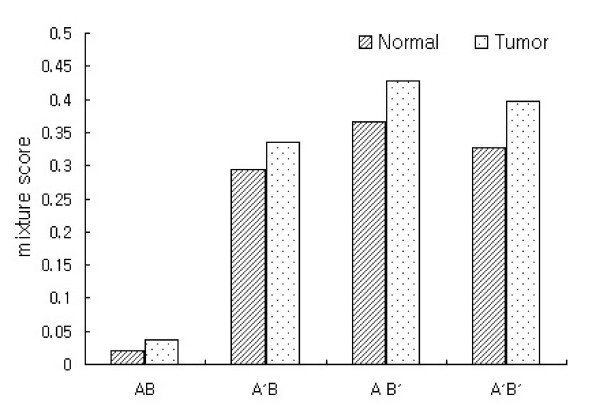
Comparison of *mixture score*. Pearson correlation coefficient was used as a similarity measure and *k *= 35.

The degree of mixture was measured by the *mixture score*. Euclidean distance and Pearson correlation coefficient were used as similarity measures to calculate the mixture score, and *k *was considered from 5 to 35 as a number of nearest neighbors (NNs). From the mixture scores before transformation, we found that the normal groups of data sets A and data set B were hardly intermixed (Figure 9). On the other hand, the tumor groups from the two data sets tended to be slightly intermixed, as the number of NNs increased before transformation. After transformation, the mixture scores were increased up to 42.9% in the tumor group as the number of NNs increased, suggesting that two data sets were well intermixed. In addition, these values were similar in Euclidean distance (data not shown) and Pearson correlation coefficient. Dataset AB' had the highest within-group correlation indicating that strong homogeneity exists in combined data set as shown in Figure 7, and its mixture score also was the largest as shown in Figure 9. Dataset A'B and A'B' were well intermixed although AB' had the highest mixture score and there were not considerable differences among them. Also, all of the proposed transformation methods were more effective in the tumor group than in the normal group, showing tumor groups were intermixed better than normal groups even before transformation. This can be interpreted that the characteristics of the original data set were preserved in the integrated data set after transformation.

### Comparison of significant gene sets selected by transformation methods

Table 1 shows the number of differently expressed genes using t-test for each transformation method. Adjusted p-values of 0.05 and 0.01 by Bonferroni correction were used as the significant levels.

As shown in Table 1, a larger number of significant genes were identified in data set AB' compared to other data sets. With the adjusted p-value of 0.01, 3868 and 2488 genes were identified by the AB' and AB method, respectively. This can be interpreted that data set AB' had large t-statistics due to the large mean differences and small variations in expression levels between two experimental groups with strong homogeneity. In ANOVA model excluding interaction term, 3004 significant genes were detected and it showed that the performance of ANOVA model is better than the use of combined data set prior to transformation, AB. However, its performance was lower than data sets transformed by our method, A'B, AB' and A'B', in comparison of the number of significant genes.

**Table 1:** Comparison of the numbers of significant genes.

**Data set (method)/α**	**0.05**	**0.01**
A	2325	2005
B	1654	1367
AB	2848	2488
A'B	3429	3088
AB'	4257	3868
A'B'	3453	3102
ANOVA	3337 (3302)	3004 (2961)

α: significant level by Bonferroni correction

The number in the parenthesis is the number of significant genes when ANOVA model included the interaction term.

We compared the degree of concurrency in top 500 significant genes which are selected from combined data sets. Two hundred fifty genes out of top 500 genes selected from data set A were consistent with gene set selected from data set B. Among 750 genes, which is union gene set of top 500 genes of data set A and data set B, 496, 492, and 488 genes were consistent with top 500 genes of A'B, AB' and A'B', respectively. And 457 genes were consistent with top 500 genes of AB, indicating that our transformation methods preserved the original biology of the data sets.

Eight genes were detected which belonged to AB' but A or B. We investigated the relationship of these genes with colorectal cancer and summarized the annotations of these genes in Table 2. Chromosomal location 3q21-q24 was known as colorectal cancer (CRC) susceptible area [16] and 4q33-q34 was identified as common genomic alteration in CRC and genomic regions with altered DNA copy numbers [17]. It is reported that biallelic germline mutations in MYH are associated with colorectal neoplasm [18], MYH111, an isoform of MYH, could be suggested as also related with colorectal cancer. Some genes among 8 genes were colon cancer related genes or located on close to a gene which is a colon cancer susceptible gene. Therefore, we confirmed that the proposed method could detect informative genes which might be lost by using separated data sets.

**Table 2:** Descriptions of 8 genes which were selected from AB' but A or B.

**Gene ID**	**UniGene ID**	**Symbol**	**Gene name**	**Chromosomal Location**
AI972269	Hs.556600	MYLK	Myosin, light chain kinase	3q21
AA447632	Hs.75819	GPM6A	Glycoprotein M6A	4q34
AA485871	Hs.286226	MYO1C	Myosin IC	17p13
AI266457	Hs.527860		Transcribed locus	12
AI383497	Hs.189409	FNBP1	Formin binding protein 1	9q34
AA463926	Hs.444403	PPP1R12B	Protein phosphatase 1, regulatory (inhibitor) subunit 12B 1q32.1	1q32.1
AI524093	Hs.460109	MYH11	Myosin, heavy chain 11, smooth muscle	16p13.11
AA213816	Hs.369574	CDC42EP3	CDC42 effector protein (Rho GTPase binding) 3	2p21

We performed the simulation study using the arbitrary data sets with various sample size, from 5 to 30, were sampled from original data set. It was processed as following.

(1) Take random subsamples from data set A and data set B (such as 5 tumor and 5 normal tissues from each set) without replacement. This process was repeated 10 times with same sample size for reducing sampling bias.

(2) Find the 500 significant genes list from the subsample of A.

(3) Find the 500 significant genes list from (subsample A)(subsample B) ', which is a combined data set with subsample of A and transformed data set of subsample of B. (4) Compare (2) and (3) with 500 significant genes list selected from data set A.

**Figure 10:**
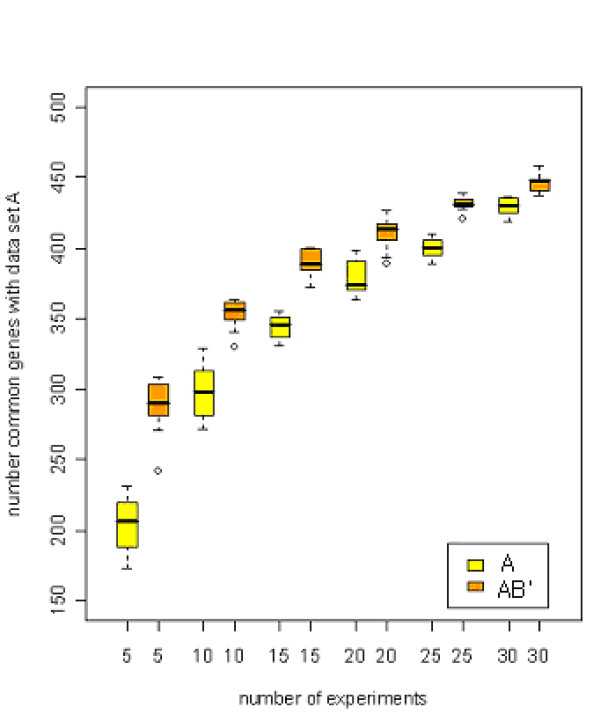
Comparison of the concurrency of significant gene sets. The vertical and horizontal axes represent the number of sampled experiments and the number of common significant genes, respectively. In legend, A and AB' represent subsample of A and a combined data set of subsample A and transformed subsample B, respectively.

Figure 10 showed that the usage of a combined data set is more effective than separated data set and the proposed method was seen more useful in a small data set. For example, when the sample size is 5, the concurrency of significant genes with those of data set A was increased rapidly by using a combined data. The concurrency was increased as the number of samples was increased.

The gene sets with a similar OOB (out-of-bag) error rate were compared (information of each gene set not shown), and none of the gene sets had 0% OOB error regardless of the transformation methods. Only two significant genes of data set AB' had 0.65% OOB error. Even if the number of significant genes was increased to 500, the OOB error rate did not decrease. The prediction accuracies of top 500 significant genes were compared, and the prediction accuracies were 100% for all of transformation methods (data not shown). In this case, integrated data sets by each transformation method were used as training data sets to create classifiers, and separated data sets were used for testing these classifiers.

## Discussion

An inescapable problem with combining several microarray data sets is the variation of expressions between data sets. In cases where the microarray analyses are from different experimental conditions, integration without transformation may skew the expression ratios of the same genes from different data sets. When the experimental bias exceeds biological variation, the use of microarray data sets without adjustments for this bias may make biological variation unidentifiable, meaning that reliable results cannot be obtained. In addition, due to the limited numbers of available microarray experiments, the motivation to use the whole data set, regardless of platforms or experimental procedure, is increasing.

We attempted to minimize experimental bias by transforming the expression ratios of the data sets such that they have similar expression patterns in the corresponding experimental groups of different data sets. Compared with previous studies, the proposed transformation method is a relatively simple algorithm [11-13], furthermore that showed good performance in various evaluation methods. While a previous study used a reference sample with average expression of whole experiments including normal and tumor groups [14], the proposed method considers biological differences that can be existed between different experimental groups by transforming expression ratios for each experimental group separately.

Our method transforms expression ratios by three approaches. In A'B, data set B was used as a reference data set, data set A in AB'. A'B' is a combined data set after transforming both of two data sets using pooled standard deviation. In selecting the reference data set, we did not consider biological meaning and we rather compared the effects of transformation with diverse references. When a method used a data set with strong homogeneity as a reference data set, its performance was slightly better than other transformation methods as shown in Figure 9, but there were no significant differences in efficiencies among them, thus allowing a biological evaluation of the significant genes of data sets integrated by each transformation method.

Using a data set with homogeneity as a reference data set, we observed that such characteristics are preserved in the combined data set more strongly. Further, two separated data sets can be well intermixed in a combined data set because there are more chances that *k *NNs of a experiment in one data set includes experiments of the other data set (AB' in Figure 9). Also, the mean difference of expressions between two experimental groups can be larger as the homogeneity increases within the group. Therefore, larger number of significant genes can be selected from AB' as shown in Table 1.

RNA source effect in gene expression ratios was detected in more than 5000 genes before transformation and such effect was adjusted by the proposed methods.

Integration of datasets increases the sample size and improves the analytical accuracy and statistical power of the test. When focusing on significant gene selection, ANOVA can be a flexible model to consider any clinical information as well as genetic information with several effect factors representing experimental conditions [15]. However, this method is applied to each gene and does not create a combined data set for applying various statistical methods to be able to identify additionally useful biological information, such as gene expression patterns through whole gene set. Therefore, for a given experimental question, i.e. complex genetic information including expression patterns, it is useful to integrate data sets by transformation prior to specific analysis.

The proposed integration method preserves the expression patterns of two data sets similar in corresponding experimental groups, transforming the location and the scale of the expression ratios and this method is available to any data set with more than two groups. We confirmed that the transformed data sets obtained from different experimental environments were well intermixed, meaning that the experimental bias was reduced. And most genes among top 500 genes, which were selected from combined data sets after transformation, were consistent with top 500 genes selected from two original data sets. This means that our method preserves original biology of two data sets. In addition, we detected colorectal cancer related genes which might be dropped in separated data sets by using a combined data set. By simulation study, we confirmed that the proposed method can detect more reliable information from a combined data and it is more effective in small data sets derived from different experimental conditions.

## Conclusion

This method may not be appropriate when the different experimental features in data sets include biological variations (for example, early disease stages of I and II in data set A and advanced disease stages of III and IV in data set B) because the expression values of a specific experimental group are transformed into the form of the corresponding experimental group of a reference data set. Thus, we suggest that the proposed integration method is useful when each data set includes phenotypically or biologically homogenous experimental groups.

In conclusion, our method is simple and useful to combine several datasets experimented under different experimental conditions and available to any data set including more than two groups. With this method, biologically useful information can be detectable by applying various analytic methods to combined data set with increased sample size.

## Methods

### Tissue sample preparation

A total of 154 colorectal tissue samples (82 tumor and 72 normal) were obtained from colorectal cancer patients who had undergone surgery at the Severance Hospital, Yonsei University College of Medicine, Seoul, Korea. Informed consent was obtained from patients prior to using their surgical specimens and clinicopathologic data for research purposes. Fresh tissues obtained from patients were snap-frozen and stored at -80°C.

### Microarrays

Total RNA was extracted from the tissues using Trizol (Invitrogen, USA) and then purified using an RNeasy kit (Qiagen, Germany). The purified RNA samples were divided into two groups for gene expression profiling using total RNA and amplified mRNA. Gene expression profiling using total RNA samples consisted of 20 paired normal and tumor colon tissue samples, 23 tumor samples, and 15 normal colon tissues. This data set is used by data set A in this study. Of the remaining 34 paired samples, 5 tumor and 3 normal colon tissues were used for gene expression profiling with amplified mRNA, which was obtained using the linear T7 mRNA amplification method with the Megascript T7 kit (Ambion, USA). This data set is used by data set B in this study. Each sample of total RNA (50 ug) and amplified mRNA (2 ug) was directly labeled with Cy5-dUTP and transcribed to cDNA. The microarray experiment was performed using a reference design with the Cy-3 dUTP labeled Yonsei reference RNA [19]. We used the 17K human cDNA microarray (GenomicTree Co., Daejon, Korea) for probe hybridization based on the Yonsei Cancer Metastasis Research Center (CMRC, Yonsei University, Korea) protocol [19]. Following hybridization, microarrays were scanned using a GenePix 4000B (Axon Ins., USA) and images were analyzed using GenePix Pro 4.0 (Axon Ins., USA).

These two microarray data sets have only difference on RNA source. Previous studies have concluded that it is vital to use equally treated samples for any particular study, and all other samples should be amplified when one sample requires amplification. In addition, the sensitivity to detect differential gene expression from microarray data set using amplified RNA was also different compared to using total RNA [20,21]. Therefore, we used these two data sets for evaluating our method.

### Data normalization

Expression intensities were normalized such that they would have similar distributions across a series of arrays. In this study, the MAD (median-absolute-deviation) scale estimator was used as a robust estimate of scale, and both A-values, as well as the M-values, were normalized. Within-slide and between slide normalization were used to transform expression values to make intensities consistent within each array and transform expression values to achieve consistency between arrays, respectively. It was necessary to apply between-slide normalization to the expression data because there were different dispersions between arrays after within-slide normalization. The normalization process was executed using the 'limma' library of the R package [22].

### Data transformation for combining data sets

The gene expression intensities of each data set were transformed based on the reference data set by the following three different methods to have similar expression patterns in corresponding experimental group.

***1) A'B***: The gene expression ratios of data set A were transformed into the form of data set B which is considered as a reference data set. The transformed expression ratios of normal and tumor groups in data set A were calculated for each gene as follows:

AN/=AN(sd(BN)/sd(AN))-[AN(sd(BN)/sd(AN))¯-BN¯)]AT/=AT(sd(BT)/sd(AT))-[AT(sd(BT)/sd(AT))¯-BT¯)]
 MathType@MTEF@5@5@+=feaafiart1ev1aaatCvAUfKttLearuWrP9MDH5MBPbIqV92AaeXatLxBI9gBaebbnrfifHhDYfgasaacH8akY=wiFfYdH8Gipec8Eeeu0xXdbba9frFj0=OqFfea0dXdd9vqai=hGuQ8kuc9pgc9s8qqaq=dirpe0xb9q8qiLsFr0=vr0=vr0dc8meaabaqaciaacaGaaeqabaqabeGadaaakeaafaqaaeGabaaabaGaemyqaeKaemOta40aaWbaaSqabeaacqGGVaWlaaGccqGH9aqpcqWGbbqqcqWGobGtcqGGOaakcqWGZbWCcqWGKbazcqGGOaakcqWGcbGqcqWGobGtcqGGPaqkcqGGVaWlcqWGZbWCcqWGKbazcqGGOaakcqWGbbqqcqWGobGtcqGGPaqkcqGGPaqkcqGGTaqlcqGGBbWwdaqdaaqaaiabdgeabjabd6eaojabcIcaOiabdohaZjabdsgaKjabcIcaOiabdkeacjabd6eaojabcMcaPiabc+caViabdohaZjabdsgaKjabcIcaOiabdgeabjabd6eaojabcMcaPiabcMcaPaaacqGGTaqldaqdaaqaaiabdkeacjabd6eaobaacqGGPaqkcqGGDbqxaeaacqWGbbqqcqWGubavdaahaaWcbeqaaiabc+caVaaakiabg2da9iabdgeabjabdsfaujabcIcaOiabdohaZjabdsgaKjabcIcaOiabdkeacjabdsfaujabcMcaPiabc+caViabdohaZjabdsgaKjabcIcaOiabdgeabjabdsfaujabcMcaPiabcMcaPiabc2caTiabcUfaBnaanaaabaGaemyqaeKaemivaqLaeiikaGIaem4CamNaemizaqMaeiikaGIaemOqaiKaemivaqLaeiykaKIaei4la8Iaem4CamNaemizaqMaeiikaGIaemyqaeKaemivaqLaeiykaKIaeiykaKcaaiabc2caTmaanaaabaGaemOqaiKaemivaqfaaiabcMcaPiabc2faDbaaaaa@8CDA@

where *AN'*, *AT'*: transformed expression ratios of normal and tumor groups in data set A.

*AN*, *AT*: normal and tumor groups in data set A.

BN¯
 MathType@MTEF@5@5@+=feaafiart1ev1aaatCvAUfKttLearuWrP9MDH5MBPbIqV92AaeXatLxBI9gBaebbnrfifHhDYfgasaacH8akY=wiFfYdH8Gipec8Eeeu0xXdbba9frFj0=OqFfea0dXdd9vqai=hGuQ8kuc9pgc9s8qqaq=dirpe0xb9q8qiLsFr0=vr0=vr0dc8meaabaqaciaacaGaaeqabaqabeGadaaakeaadaqdaaqaaiabdkeacjabd6eaobaaaaa@2EEF@, BT¯
 MathType@MTEF@5@5@+=feaafiart1ev1aaatCvAUfKttLearuWrP9MDH5MBPbIqV92AaeXatLxBI9gBaebbnrfifHhDYfgasaacH8akY=wiFfYdH8Gipec8Eeeu0xXdbba9frFj0=OqFfea0dXdd9vqai=hGuQ8kuc9pgc9s8qqaq=dirpe0xb9q8qiLsFr0=vr0=vr0dc8meaabaqaciaacaGaaeqabaqabeGadaaakeaadaqdaaqaaiabdkeacjabdsfaubaaaaa@2EFB@: mean expression ratios of tumor and normal groups in data set B.

*sd*(*AN*), *sd*(*AT*), *sd*(*BN*), *sd*(*BT*): standard deviation of expression ratios of tumor and normal groups in data set A and B.

***2) AB'***: The gene expression ratios of data set B were transformed into the form of data set A which is considered as a reference data set. The transformed expression ratios of normal and tumor groups in data set B were calculated for each gene as follows:

BN/=BN(sd(AN)/sd(BN))-[BN(sd(AN)/sd(BN))¯-AN¯)]BT/=BT(sd(AT)/sd(BT))-[BT(sd(AT)/sd(BT))¯-AT¯)]
 MathType@MTEF@5@5@+=feaafiart1ev1aaatCvAUfKttLearuWrP9MDH5MBPbIqV92AaeXatLxBI9gBaebbnrfifHhDYfgasaacH8akY=wiFfYdH8Gipec8Eeeu0xXdbba9frFj0=OqFfea0dXdd9vqai=hGuQ8kuc9pgc9s8qqaq=dirpe0xb9q8qiLsFr0=vr0=vr0dc8meaabaqaciaacaGaaeqabaqabeGadaaakeaafaqaaeGabaaabaGaemOqaiKaemOta40aaWbaaSqabeaacqGGVaWlaaGccqGH9aqpcqWGcbGqcqWGobGtcqGGOaakcqWGZbWCcqWGKbazcqGGOaakcqWGbbqqcqWGobGtcqGGPaqkcqGGVaWlcqWGZbWCcqWGKbazcqGGOaakcqWGcbGqcqWGobGtcqGGPaqkcqGGPaqkcqGGTaqlcqGGBbWwdaqdaaqaaiabdkeacjabd6eaojabcIcaOiabdohaZjabdsgaKjabcIcaOiabdgeabjabd6eaojabcMcaPiabc+caViabdohaZjabdsgaKjabcIcaOiabdkeacjabd6eaojabcMcaPiabcMcaPaaacqGGTaqldaqdaaqaaiabdgeabjabd6eaobaacqGGPaqkcqGGDbqxaeaacqWGcbGqcqWGubavdaahaaWcbeqaaiabc+caVaaakiabg2da9iabdkeacjabdsfaujabcIcaOiabdohaZjabdsgaKjabcIcaOiabdgeabjabdsfaujabcMcaPiabc+caViabdohaZjabdsgaKjabcIcaOiabdkeacjabdsfaujabcMcaPiabcMcaPiabc2caTiabcUfaBnaanaaabaGaemOqaiKaemivaqLaeiikaGIaem4CamNaemizaqMaeiikaGIaemyqaeKaemivaqLaeiykaKIaei4la8Iaem4CamNaemizaqMaeiikaGIaemOqaiKaemivaqLaeiykaKIaeiykaKcaaiabc2caTmaanaaabaGaemyqaeKaemivaqfaaiabcMcaPiabc2faDbaaaaa@8CE2@

where *BN'*, *BT'*: transformed expression ratios of normal and tumor groups in data set B.

*BN*, *BT*: normal and tumor groups in data set B.

AN¯
 MathType@MTEF@5@5@+=feaafiart1ev1aaatCvAUfKttLearuWrP9MDH5MBPbIqV92AaeXatLxBI9gBaebbnrfifHhDYfgasaacH8akY=wiFfYdH8Gipec8Eeeu0xXdbba9frFj0=OqFfea0dXdd9vqai=hGuQ8kuc9pgc9s8qqaq=dirpe0xb9q8qiLsFr0=vr0=vr0dc8meaabaqaciaacaGaaeqabaqabeGadaaakeaadaqdaaqaaiabdgeabjabd6eaobaaaaa@2EED@, AT¯
 MathType@MTEF@5@5@+=feaafiart1ev1aaatCvAUfKttLearuWrP9MDH5MBPbIqV92AaeXatLxBI9gBaebbnrfifHhDYfgasaacH8akY=wiFfYdH8Gipec8Eeeu0xXdbba9frFj0=OqFfea0dXdd9vqai=hGuQ8kuc9pgc9s8qqaq=dirpe0xb9q8qiLsFr0=vr0=vr0dc8meaabaqaciaacaGaaeqabaqabeGadaaakeaadaqdaaqaaiabdgeabjabdsfaubaaaaa@2EF9@: mean expression ratios of tumor and normal groups in data set A.

***3) A'B'***: The gene expression ratios of data set A and data set B were transformed using the pooled standard deviation and mean expression values of the two data sets. The transformed expression ratios of the normal and tumor groups in data set A and data set B were calculated for each gene as follows:

AN/=AN(sd(N))/sd(AN)-(AN(sd(N))/sd(AN)¯-N¯)AT/=AT(sd(T))/sd(AT)-(AT(sd(T))/sd(AT)¯-T¯)
 MathType@MTEF@5@5@+=feaafiart1ev1aaatCvAUfKttLearuWrP9MDH5MBPbIqV92AaeXatLxBI9gBaebbnrfifHhDYfgasaacH8akY=wiFfYdH8Gipec8Eeeu0xXdbba9frFj0=OqFfea0dXdd9vqai=hGuQ8kuc9pgc9s8qqaq=dirpe0xb9q8qiLsFr0=vr0=vr0dc8meaabaqaciaacaGaaeqabaqabeGadaaakeaafaqaaeGabaaabaGaemyqaeKaemOta40aaWbaaSqabeaacqGGVaWlaaGccqGH9aqpcqWGbbqqcqWGobGtcqGGOaakcqWGZbWCcqWGKbazcqGGOaakcqWGobGtcqGGPaqkcqGGPaqkcqGGVaWlcqWGZbWCcqWGKbazcqGGOaakcqWGbbqqcqWGobGtcqGGPaqkcqGGTaqlcqGGOaakdaqdaaqaaiabdgeabjabd6eaojabcIcaOiabdohaZjabdsgaKjabcIcaOiabd6eaojabcMcaPiabcMcaPiabc+caViabdohaZjabdsgaKjabcIcaOiabdgeabjabd6eaojabcMcaPaaacqGGTaqldaqdaaqaaiabd6eaobaacqGGPaqkaeaacqWGbbqqcqWGubavdaahaaWcbeqaaiabc+caVaaakiabg2da9iabdgeabjabdsfaujabcIcaOiabdohaZjabdsgaKjabcIcaOiabdsfaujabcMcaPiabcMcaPiabc+caViabdohaZjabdsgaKjabcIcaOiabdgeabjabdsfaujabcMcaPiabc2caTiabcIcaOmaanaaabaGaemyqaeKaemivaqLaeiikaGIaem4CamNaemizaqMaeiikaGIaemivaqLaeiykaKIaeiykaKIaei4la8Iaem4CamNaemizaqMaeiikaGIaemyqaeKaemivaqLaeiykaKcaaiabc2caTmaanaaabaGaemivaqfaaiabcMcaPaaaaaa@833C@

BN/=BN(sd(N))/sd(BN)-(BN(sd(N))/sd(BN)¯-N¯)BT/=BT(sd(T))/sd(BT)-(BT(sd(T))/sd(BT)¯-T¯)
 MathType@MTEF@5@5@+=feaafiart1ev1aaatCvAUfKttLearuWrP9MDH5MBPbIqV92AaeXatLxBI9gBaebbnrfifHhDYfgasaacH8akY=wiFfYdH8Gipec8Eeeu0xXdbba9frFj0=OqFfea0dXdd9vqai=hGuQ8kuc9pgc9s8qqaq=dirpe0xb9q8qiLsFr0=vr0=vr0dc8meaabaqaciaacaGaaeqabaqabeGadaaakeaafaqaaeGabaaabaGaemOqaiKaemOta40aaWbaaSqabeaacqGGVaWlaaGccqGH9aqpcqWGcbGqcqWGobGtcqGGOaakcqWGZbWCcqWGKbazcqGGOaakcqWGobGtcqGGPaqkcqGGPaqkcqGGVaWlcqWGZbWCcqWGKbazcqGGOaakcqWGcbGqcqWGobGtcqGGPaqkcqGGTaqlcqGGOaakdaqdaaqaaiabdkeacjabd6eaojabcIcaOiabdohaZjabdsgaKjabcIcaOiabd6eaojabcMcaPiabcMcaPiabc+caViabdohaZjabdsgaKjabcIcaOiabdkeacjabd6eaojabcMcaPaaacqGGTaqldaqdaaqaaiabd6eaobaacqGGPaqkaeaacqWGcbGqcqWGubavdaahaaWcbeqaaiabc+caVaaakiabg2da9iabdkeacjabdsfaujabcIcaOiabdohaZjabdsgaKjabcIcaOiabdsfaujabcMcaPiabcMcaPiabc+caViabdohaZjabdsgaKjabcIcaOiabdkeacjabdsfaujabcMcaPiabc2caTiabcIcaOmaanaaabaGaemOqaiKaemivaqLaeiikaGIaem4CamNaemizaqMaeiikaGIaemivaqLaeiykaKIaeiykaKIaei4la8Iaem4CamNaemizaqMaeiikaGIaemOqaiKaemivaqLaeiykaKcaaiabc2caTmaanaaabaGaemivaqfaaiabcMcaPaaaaaa@8350@

where N¯
 MathType@MTEF@5@5@+=feaafiart1ev1aaatCvAUfKttLearuWrP9MDH5MBPbIqV92AaeXatLxBI9gBaebbnrfifHhDYfgasaacH8akY=wiFfYdH8Gipec8Eeeu0xXdbba9frFj0=OqFfea0dXdd9vqai=hGuQ8kuc9pgc9s8qqaq=dirpe0xb9q8qiLsFr0=vr0=vr0dc8meaabaqaciaacaGaaeqabaqabeGadaaakeaadaqdaaqaaiabd6eaobaaaaa@2DE2@, T¯
 MathType@MTEF@5@5@+=feaafiart1ev1aaatCvAUfKttLearuWrP9MDH5MBPbIqV92AaeXatLxBI9gBaebbnrfifHhDYfgasaacH8akY=wiFfYdH8Gipec8Eeeu0xXdbba9frFj0=OqFfea0dXdd9vqai=hGuQ8kuc9pgc9s8qqaq=dirpe0xb9q8qiLsFr0=vr0=vr0dc8meaabaqaciaacaGaaeqabaqabeGadaaakeaadaqdaaqaaiabdsfaubaaaaa@2DEE@: mean expression ratios of normal and tumor groups in data set A and data set B.

sd(N)=(nAN-1)sd(AN)2+(nBN-1)sd(BN)2nAN+ nBN-2
 MathType@MTEF@5@5@+=feaafiart1ev1aaatCvAUfKttLearuWrP9MDH5MBPbIqV92AaeXatLxBI9gBaebbnrfifHhDYfgasaacH8akY=wiFfYdH8Gipec8Eeeu0xXdbba9frFj0=OqFfea0dXdd9vqai=hGuQ8kuc9pgc9s8qqaq=dirpe0xb9q8qiLsFr0=vr0=vr0dc8meaabaqaciaacaGaaeqabaqabeGadaaakeaacqWGZbWCcqWGKbazcqGGOaakcqWGobGtcqGGPaqkcqGH9aqpdaGcaaqaamaalaaabaGaeiikaGIaemOBa42aaSbaaSqaaiabdgeabjabd6eaobqabaGccqGGTaqlcqaIXaqmcqGGPaqkcqWGZbWCcqWGKbazcqGGOaakcqWGbbqqcqWGobGtcqGGPaqkdaahaaWcbeqaaiabikdaYaaakiabgUcaRiabcIcaOiabd6gaUnaaBaaaleaacqWGcbGqcqWGobGtaeqaaOGaeiyla0IaeGymaeJaeiykaKIaem4CamNaemizaqMaeiikaGIaemOqaiKaemOta4KaeiykaKYaaWbaaSqabeaacqaIYaGmaaaakeaacqWGUbGBdaWgaaWcbaGaemyqaeKaemOta4eabeaakiabgUcaRiabbccaGiabd6gaUnaaBaaaleaacqWGcbGqcqWGobGtaeqaaOGaeiyla0IaeGOmaidaaaWcbeaaaaa@5DA3@: pooled standard deviation of the normal group

sd(T)=(nAT-1)sd(AT)2+(nBT-1)sd(BT)2nAT+ nBT-2
 MathType@MTEF@5@5@+=feaafiart1ev1aaatCvAUfKttLearuWrP9MDH5MBPbIqV92AaeXatLxBI9gBaebbnrfifHhDYfgasaacH8akY=wiFfYdH8Gipec8Eeeu0xXdbba9frFj0=OqFfea0dXdd9vqai=hGuQ8kuc9pgc9s8qqaq=dirpe0xb9q8qiLsFr0=vr0=vr0dc8meaabaqaciaacaGaaeqabaqabeGadaaakeaacqWGZbWCcqWGKbazcqGGOaakcqWGubavcqGGPaqkcqGH9aqpdaGcaaqaamaalaaabaGaeiikaGIaemOBa42aaSbaaSqaaiabdgeabjabdsfaubqabaGccqGGTaqlcqaIXaqmcqGGPaqkcqWGZbWCcqWGKbazcqGGOaakcqWGbbqqcqWGubavcqGGPaqkdaahaaWcbeqaaiabikdaYaaakiabgUcaRiabcIcaOiabd6gaUnaaBaaaleaacqWGcbGqcqWGubavaeqaaOGaeiyla0IaeGymaeJaeiykaKIaem4CamNaemizaqMaeiikaGIaemOqaiKaemivaqLaeiykaKYaaWbaaSqabeaacqaIYaGmaaaakeaacqWGUbGBdaWgaaWcbaGaemyqaeKaemivaqfabeaakiabgUcaRiabbccaGiabd6gaUnaaBaaaleaacqWGcbGqcqWGubavaeqaaOGaeiyla0IaeGOmaidaaaWcbeaaaaa@5DF7@: pooled standard deviation of the tumor group

*n*_*AN*_, *n*_*BN*_, *n*_*AT*_, *n*_*BT*_: number of experiments of *AN*, *BN*, *AT *and *BT*.

### Evaluation of transformation method

We evaluated our proposed integration method by several plots and *mixture score*, defined to evaluate the efficiency of the integration method proposed in this study. The principle of this metric is to measure how many k-nearest neighbors (kNNs) of data set B in combined data set belong to data set A. The metric was calculated as follows, where *k *is the number of nearest neighbors (NNs).

***Mixture score ***= #{*x*/*x *∈ *k*NNs(data set B) n (data set A)}/*k*

where *x *is any experiment belonging to kNNs(data set B) and data set A.

The mixture score ranges from 0 to 1. A value close to 0.5 is indicative of two different data sets that are perfectly intermixed. Conversely, values close to either 0 or 1 indicate a poor level of intermixing between the two different data sets.

We evaluated the classification accuracies of selected gene sets from integrated data sets by different transformation methods and used Random Forest algorithm (RF) [23] for this. We used RF program in R package [22] with the following steps.

(1) Generate *n *datasets of bootstrap samples {*B*_1_, *B*_2_,..., *B*_*n*_} by allowing repetition of the same sample.

(2) Use each sample *B*_*k *_to construct a Tree classifier *T*_*k *_to predict those samples that are not in *B*_*k*_, called out-of-bag (OOB) samples. These predictions are called out-of-bag estimators.

(3) Final prediction is the average of out-of-bag estimators over all bootstrap samples and we get average of them which is overall classification error (OOB error).

ANOVA (Analysis of Variance) model was used to evaluate the RNA source effect of data sets derived from different experimental conditions. ANOVA model used in this work is as following.

*g*_*ijk *_= *μ *+ *T*_*i *_+ *R*_*j *_+ (*TR*)_*ij *_+ *ε*_*ijk*_, *ε*_*ijk *_~ *N*(0, *σ*^2^), *i *= 1, 2. *j *= 1, 2. *k *= 1, 2,...,154.

where *g*_*ijk *_is *k*^*th *^expression ratio of a gene in *i*^*th *^treatment and *j*^*th *^RNA source. *T*_*i*_, *R*_*j *_and (*TR*)_*ij *_represent treatment effect, RNA source effect and interaction effect, respectively.

## Abbreviations

SVD: Singular Value Decompositin, SVM: Support Vector Machine, KNN: K Nearest Neighbors, NNs: Nearest Neighbors, ANOVA: Analysis Of Variance, OOB error; Out Of Bag error, HCA: Hierarchical Cluster Analysis.

## Authors' contributions

KYK participated in the design of algorithms, performed statistical analysis and drafted the manuscript. DHK performed the microarray experiments with amplified RNA. HJJ carried out the microarray with total RNA. HCJ participated in getting the consent form the patients and obtained the clinical data. HCC participated in the study design and data interpretation. SYR conceived of the study, participated in its design and coordination, and finalized manuscript.
